# Labelling via [Al^18^F]^2+^ Using Precomplexed Al-NODA Moieties

**DOI:** 10.3390/ph14080818

**Published:** 2021-08-20

**Authors:** Daniel Kang, Ulrich Simon, Felix M. Mottaghy, Andreas T. J. Vogg

**Affiliations:** 1 Institute of Inorganic Chemistry, RWTH Aachen University, 52056 Aachen, Germany; daniel.kang@rwth-aachen.de (D.K.); ulrich.simon@ac.rwth-aachen.de (U.S.); 2 Department of Nuclear Medicine, Uniklinik RWTH Aachen, 52074 Aachen, Germany; fmottaghy@ukaachen.de; 3 Department of Radiology and Nuclear Medicine, Maastricht University Medical Center (MUMC+), 6202 AZ Maastricht, The Netherlands

**Keywords:** [Al^18^F]^2+^ cation, chelator labelling, reaction optimization, aqueous [^18^F]fluoride labelling

## Abstract

Over the past 20 years, ^68^Ga-labelled radiopharmaceuticals have become an important part in clinical routine. However, the worldwide supply with ^68^Ge/^68^Ga generators is limited as well as the number of patient doses per batch of ^68^Ga radiopharmaceutical. In the recent years, a new technique appeared, making use of the ease of aqueous labelling via chelators as with ^68^Ga but using ^18^F instead. This technique takes advantage of the strong coordinative bond between aluminium and fluoride, realized in the aqueous cation [Al^18^F]^2+^. Most applications to date make use of one-pot syntheses with free Al(III) ions in the system. In contrast, we investigated the labelling approach split into two steps: generating the Al-bearing precursor in pure form and using this Al compound as a precursor in the labelling step with aqueous [^18^F]fluoride. Hence, no free Al^3+^ ions are present in the labelling step. We investigated the impact of parameters: temperature, pH, addition of organic solvent, and reaction time using the model chelator NH_2_-MPAA-NODA. With optimized parameters we could stably achieve a 80% radiochemical yield exerting a 30-min reaction time at 100 °C. This technique has the potential to become an important approach in radiopharmaceutical syntheses.

## 1. Introduction

Over the recent decades, nuclear molecular imaging (especially positron emission tomography, PET) has emerged as a game changer in oncologic management using a broad variety of radiolabelled probes, also called radiotracers. Among these radiotracers, receptor-directed radiolabelled peptides—e.g., [^68^Ga]Ga-DOTATOC—became widely used as radiopharmaceuticals in specific malignancies [1,2]. Dependent on the application, today, there is an expanding range of radionuclides available, hence making nuclear medicine adaptive and highly versatile [3,4]. To date, PET-CT scanners deliver optimal image qualities based on the commonly used positron emitters ^18^F, ^68^Ga, and ^11^C [5,6]. Regarding the choice of the appropriate nuclide, its half-life, emission characteristics and availability play a major role. While ^11^C requires a costly cyclotron facility on site, ^18^F can be supplied by a satellite infrastructure, if available [7]. In contrast, radionuclide generators allow daily access to nuclides even if the daily supply infrastructure is weak. Thus, with the introduction of ^68^Ga generators about 20 years ago, PET became available on every medical site, while the costs per GBq initially were reasonable. The primary advantage of ^68^Ga from generators—besides its steady presence in the laboratory—is the ease of its labelling procedure [8,9]. The nuclide forms a stable complex with chelators, such as 1,4,7-triazacyclononane-1,4,7-triacetic acid (NOTA) or 1,4,7,10-tetraazacyclododecane-1,4,7,10-tetracetic acid (DOTA) [10]. These chelators can easily be linked to peptides or small molecules, which allows ^68^Ga labelling of almost any substrate. Since the complex formation at the optimal pH is robust and can be achieved quantitatively within minutes, this labelling approach gained broad application both in research and routine production [11,12]. Standard labelling procedures with ^18^F, in contrast, are more complex when covalent C-F bonds are aimed at, as in the labelling process of [^18^F]fluorodeoxyglucose ([^18^F]FDG) [13,14]. Over the last ten years, attempts were made to combine the ease of labelling via complexes with the favorable nuclide properties of ^18^F (t_1/2_ = 109.7 min, ∼97% β^+^ emission, low 0.635 MeV β^+^ energy) [15,16,17]. Moreover, nowadays, ^18^F can be produced by cyclotrons in high activities in the range of 100 GBq, while ^68^Ga generators usually provide 2 GBq per elution only, paired with a significantly shorter export range of respective radiopharmaceuticals due to the limited half-life [8,18]. Nevertheless, ^68^Ga radiopharmaceuticals gained practical importance with increasing demands worldwide, resulting in shortages in the supply and much increased costs for medically approved ^68^Ga generators [19].

**Figure 1:**
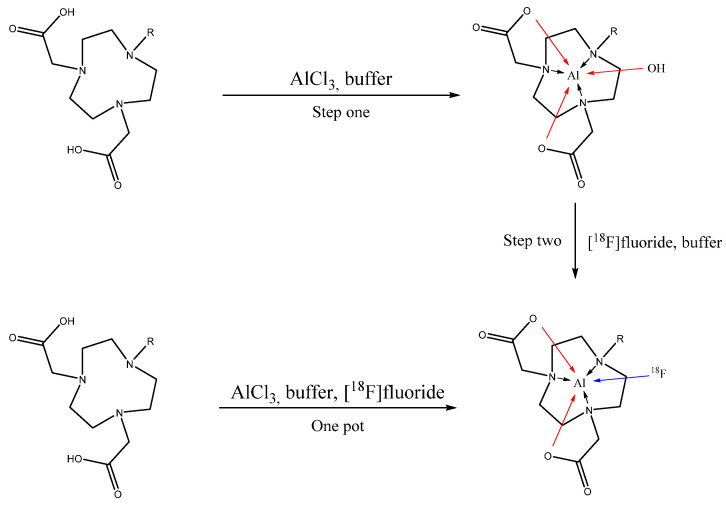
Two possible ways of forming [^18^F][AlF(NODA)] complexes. Outlined below is the established one-pot reaction [15,16,22], above our approach including the formation of the purified [Al(OH)(NODA)] complex first, followed by ligand exchange of OH^-^ against [^18^F]fluoride.

Hence, replacement of ^68^Ga by ^18^F can result in a significant cost reduction paired with a reliable supply [20]. Labelling of peptides with [^18^F]fluoride has so far required protection group chemistry or labelling via prosthetic groups [21]. A decade ago, McBride et al., followed by Laverman et al. [15,16,22], initially reported a technique that exploits the fluorophilic nature of aluminum to afford direct aqueous ^18^F-labelling by the formation of stable aluminum fluoride chelatic bonds. Their method of radiolabelling was carried out by predominantly applying a one-pot fluorination process, which yielded sufficiently stable [Al^18^F] complexes. This method of basically forming the complex cation [Al^18^F]^2+^ first, followed by coordinating this positively charged complex-ion [Al^18^F]^2+^ by the NODA moiety, in the recent years gained application due to its simplicity [23,24]. This work explores parameters and benefits of splitting this process in two steps by coordinating the aluminum cation first, followed by labelling of this Al bearing precursor with aqueous [^18^F]fluoride in a second step, thus avoiding free aluminum ions in the labelling system while aiming at likewise high labelling yields (Figure 1).

## 2. Results

### 2.1. Aluminum Coordination

**Figure 2:**
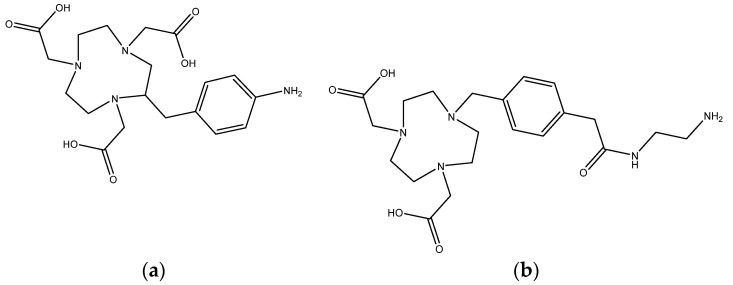
Molecular structures of chelators investigated in this study: (**a**) p-NH_2_-Bn-NOTA (termed NOTA*), and (**b**) NH_2_-MPAA-NODA (termed NODA*).

**Figure 3:**
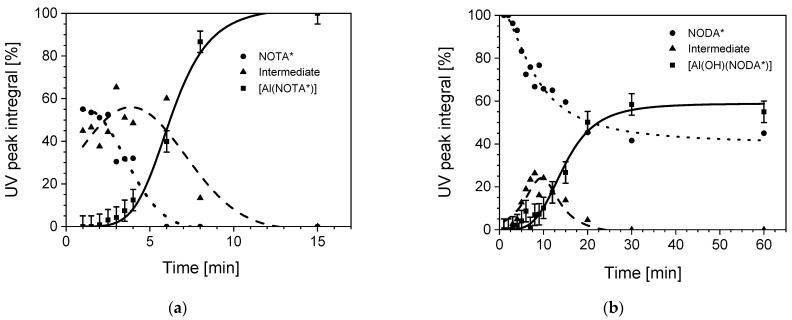
(**a**) Formation of the [Al(NOTA*)] complex over a time span of 15 min determined by HPLC measurements. Reaction conditions: 1.23-fold Al^3+^ ion excess compared to chelator, NOTA* concentration of 1.84 mM, 55 mM of NH_4_OAc-buffer pH 4.1, reaction temperature of 100 °C. Aqueous reaction medium. UV detection at 254 nm. Retention times were 2.2, 2.9, and 5.7 for the species: [Al(NOTA*)], intermediate and NOTA* (precursor), respectively (**b**) Formation of [Al(OH)(NODA*)] complex over a time span of 60 min determined by HPLC measurements. Reaction parameters and detection were analogous to the reaction with the NOTA* chelator. Only buffer concentration was higher: 91 mM. Retention times were 5.4, 6.0, and 6.9 for the species: intermediate, [Al(OH)(NODA*)], and NODA* (precursor), respectively. For better legibility in both plots, error bars (±5%) are only shown for the product data.

The reaction of 1,4,7-triazacyclononane-1,4-diacetic acid (NODA) or 1,4,7-triazacyclononane-1,4,7-triacetic acid (NOTA) with Al^3+^ was studied over time using simple derivatives NH_2_-MPAA-NODA (further termed NODA*) and p-NH_2_-Bn-NOTA (further termed NOTA*) to characterize their ability to form complexes with the metal ion. The primary difference between NODA and NOTA is their denticity, cf. their molecular structures in Figure 2. While both systems contribute three tertiary amine groups to the formation of the complex, NODA* is lacking one carboxylic acid, resulting in the need for an additional ligand to form stable octahedral complexes with Al^3+^. This leads to a saturated coordination of aluminum by NOTA* compared to NODA* where an additional ligand, such as a hydroxide group, is needed. According to Figure 3a, the coordination of aluminum with NOTA* was completed quantitatively after 15 min at 100 °C, applying a 1.23-fold molar excess of Al^3+^ ions, with no metal free chelator detectable. In this process, an intermediate appeared, which was absent when the reaction was completed. The intermediate compound may be a complex of Al^3+^ with only three carboxylic functions of the NOTA* chelator and further monodentate ligands, such as water, OH^−^, or Cl^−^. A comparison of the kinetics of both complexes with Al(III) shows a significantly slower reaction of aluminum with NODA* (Figure 3b). An equilibrium obviously limits yields at 60%, thus making longer reaction times needless. Furthermore, the formation of an intermediate was observed which also disappears during the formation process of the product. 

### 2.2. [^18^F] Fluoride Labelling

While NOTA* showed a higher ability of binding aluminum, we could achieve almost no subsequent labelling with [^18^F]fluoride. This is most likely due to the chelate effect through intramolecular complexation and thereby full (octahedral) coordination of the Al-center by the three adjacent carboxylate groups. Hence, although one might assume that, according to Pearson’s hard and soft acids and bases (HSAB) concept, F^−^—i.e., a hard base—has a significantly higher binding affinity to Al^3+^—i.e., a hard acid—, the chelate effect overcompensates the expected more stable pair formation between Al^3+^ and F^−^. 

Hence, we further focused our investigations on NODA* and all following results were received with this chelator.

#### 2.2.1. Solvent Dependency

As it was reported for one-pot reactions, the addition of organic solvents can increase [16] the radiochemical yield (RCY), so we investigated the influence of mixtures of water with some mixable polar organic solvents without losing solubility of the complexes. 

**Figure 4:**
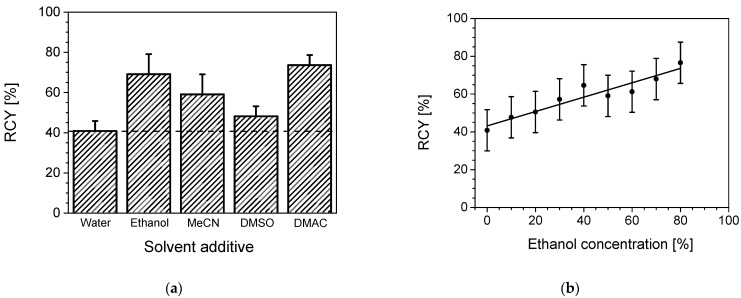
(**a**) Influence of different solvents substitutes on the yield of ^18^F labelling. Fifty per cent of the reaction solvent water were replaced by the following solvents: ethanol, acetonitrile, dimethylsulfoxide (DMSO), and N,N-dimethylacetamide (DMAC). Reaction parameters: 0.2 mM [Al(OH)(NODA*)], 0.15 M of NH_4_OAc-buffer pH 4.5, 105 °C, total volume of 100 µL with water/solvent = 50:50, 20-min reaction time. Error bars are only shown in the up direction. (**b**) Influence of the concentration of the chosen solvent ethanol (in % of total volume) in water on the RCY. Reaction parameters: 0.2 mM [Al(OH)(NODA*)], 1.5 M of NH_4_OAc-buffer pH 4.5, 105 °C, 20-min reaction time, total volume of 100 µL, 80 µL thereof with different EtOH/water ratios (*v*/*v*). The Pearson correlation coefficient is R = 0.9481.

All tests were executed with a water to solvent ratio of 1:1. As shown in Figure 4a, the RCY dependence on the solvent ranged from 40 to 73%. Replacing 50% of the water content during the reaction by acetonitrile resulted in an about 30% increase in RCY. The highest yields, however, were achieved with ethanol (69.1%) and DMAC (73.6%), respectively. While DMAC showed the best results regarding RCY, it seemed to interfere with the product. HPLC analyses showed the formation of multiple peaks close to the retention time of the labelled chelate. Possibly DMAC took part in the coordination process via the free electron pair of the nitrogen. 

Regarding a decision for an optimal solvent addition, besides solvability of all components, the solvent shall not act as a complex ligand and ideally exhibits minimum toxicity.

#### 2.2.2. Ethanol Concentration Dependency 

We further investigated the impact of the concentration or fraction of organic solvent ethanol within the aqueous reaction medium. In comparison with a purely water-based reaction, an 80% ethanol content nearly doubles the RCY from 40.8% to 76.6%, as shown in Figure 4b. The variance between the repeated reactions was quite high. Yields of the same reaction differed up to 25%. This may be explained by the applied experimental small reaction volumes and the not 100 % tight sealing of the reactor. The organic component, in particular, might be lost to some extent, thus changing the composition during the reaction. Nevertheless, a clear trend of increasing RCY with increasing EtOH content in the reaction mixture is observable (Figure 4b).

#### 2.2.3. Temperature Dependency 

**Figure 5:**
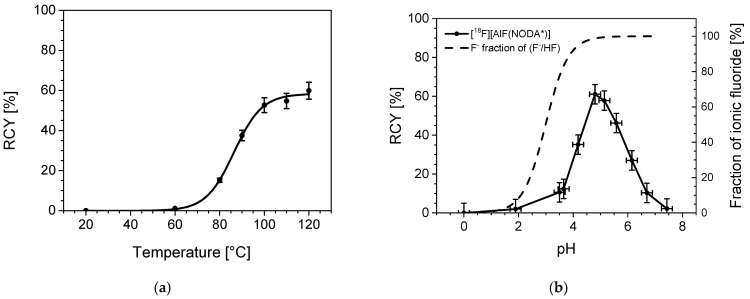
(**a**) Temperature dependency of ^18^F labelling of [Al(OH)(NODA*)]. Reaction parameters: 0.2 mM [Al(OH)(NODA*)], 0.15 M NH_4_OAc buffer pH of 4.5, total volume of 100 µL with water/ethanol = 50:50, 20-min reaction time. (**b**) Influence of the pH on [^18^F]fluoride coordination to [Al(OH)(NODA*)]. Reaction parameters: 0.2 mM [Al(OH)(NODA*)], 0.15 M of NH_4_OAc-buffer, pH of <3 by addition of HCl, 105 °C, total volume of 100 µL, solvent: water/ ethanol = 50:50, 20-min reaction time. Shown also in the graph is the corresponding fraction of ionic fluoride among the two species HF and fluoride. This function is calculated based on pKa = 3.2 [25].

Temperature has a big impact on the formation of the aluminum chelate; this also applies to the ^18^F-labelling. No labelling can be observed at room temperature. Formation of [^18^F][AlF(NODA*)] starts at around 60 °C. As clearly seen in Figure 5a, the highest RCY was observed at 120 °C yielding 59.9 % product, but was almost already plateaued at 110 °C with 54.8% RCY under these conditions. This reactions series was carried out with a 20-min reaction time.

#### 2.2.4. pH Dependency

Investigating the impact of pH on the RCY was executed by preparing NH_4_OAc-buffers with pH values between 0 and 8. pH 2 and 0 were achieved by the addition of HCl to HOAc. Coordination reactions are known for being very pH sensitive. This can be observed here as well, as shown in Figure 5b. In an alkaline medium (pH 7.5), [^18^F]fluoride coordination can be observed with maximum 5% RCY due to increasing concentrations of competing OH^-^ ions. At low pH values under 2, equally small RCY were observed with no observable labelling at pH zero, mainly due to [^18^F]HF being the dominant species, cf. the additional fluoride species fraction curve in Figure 5b. The highest RCYs were observed between 4.5 and 5.5 with a maximal yield of 61.1% at pH 4.8. The window of an optimal pH environment, thus, is narrow, making buffer systems mandatory. Other buffer systems such as HOAc/NaOAc or HCl/HEPES showed similar results, while citrate buffer systems do not seem to allow [^18^F]fluoride coordination at all. 

#### 2.2.5. Time Dependency: Reaction Kinetics

**Figure 6:**
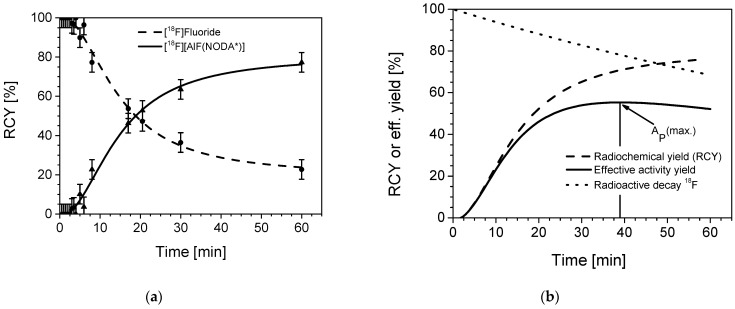
(**a**) Time dependency (kinetics) of [^18^F]fluoride exchange coordination with the [Al(OH)(NODA*)] complex according to a pseudo first order reaction, resulting in [^18^F][AlF(NODA*)] (**b**) Course of the radiochemical yield (RCY, dashed), ^18^F decay curve (dotted) and combined calculated effective activity yield (straight) over time. Reaction parameters: 0.33 mM [Al(OH)(NODA*)], 0.25 M of NH_4_OAc-buffer, pH 4.5, 105 °C, total volume of 300 µL, solvent: water/ethanol 83:17.

Previous parameters of the labelling reaction were carried out with a 20 min reaction time. To determine the kinetics of this reaction, probes were taken over a time span of 60 min, stored on ice, and subsequently analyzed by HPLC. As observed in most reactions with n.c.a. radionuclides, the product kinetics follow a pseudo-first order law with exponential saturation, while the corresponding species fraction of free [^18^F]fluoride follows an exponential decrease. As shown in Figure 6a, the maximum RCY was reached almost after 60 min with 76.2% RCY, whereas 63.6% RCY were achieved already after a 30 min reaction time. Figure 6b shows the radioactive decay of ^18^F (dotted line) together with the course of RCY (dashed line). The combination (product) of both (straight line) results in a mathematical maximum effective yield of 55.3% after a 39 min reaction time at 105 °C. 

## 3. Materials and Methods

If not specified differently, reported radiochemical yields (RCY) mean the HPLC based decay-corrected product fraction in percent of all species seen in the chromatogram of the crude reaction volume, cf. [26]. In contrast to silica-based RP phases, the polystyrene-divinylbenzene-based HPLC phase avoided losses (retention) of [^18^F]fluoride on the resin. NOTA* (p-NH_2_-Bn-NOTA, CAS no. 142131-37-1) was purchased from Macrocyclics (Plano, TX, USA), NODA* (NH_2_-MPAA-NODA) from CheMatech (Dijon, France). [^18^F]fluoride was generated by (p,n) reaction on [^18^O]water and purchased from the Research Centre Jülich, INM-5 (Jülich, Germany). The crude delivered solution was purified by passing through an anion exchange column (Sep-Pak Accell Plus QMA Plus Light cartridge, with 130 mg resin) from Waters (Milford, MA, USA), and eluted with 1 mL of saline. These [^18^F]fluoride solutions were used in all labelling experiments. An aqueous 3 M NH_4_OAc stem solution was used in all experiments. General HPLC conditions were as follows. Detection modes: Gamma (NaI) and UV (254 nm). Gradient: 1–19 min: 100% 0.1 M TFA aq → 100% MeCN. Column: PRP-1-10^#^ (250 × 4 mm, CS-Chromatographie Service, Langerwehe, Germany), flow: 2 mL/min. ^#^) Resin was from HAMILTON (Reno, NV, USA).

### 3.1. Snythesis of Aluminum Chelates

#### 3.1.1. Synthesis with the NOTA Chelator

Reaction conditions: 600 nmol of p-NH_2_-Bn-NOTA (in 300 μL water) reacted with 740 nmol of AlCl_3_ in a 0.05 M HCl solution (20 μL) plus 6 μL of NH_4_OAc-buffer 3 M. Reaction pH was 4.1 and the reaction was maintained at 100 °C for 20 min. The product was purified via HPLC. Experiments were performed in duplicate (*n* = 2).

#### 3.1.2. Synthesis with the NODA Chelator

##### Time Dependency (Kinetics)

Reaction conditions: 600 nmol of NODA* (in 300 μL water) reacted with 740 nmol of AlCl_3_ in a 0.05 M HCl solution (20 μL) plus 10 μL of NH_4_OAc-buffer 3 M. Reaction pH was 4.1 and the reaction was maintained at 100 °C for 20 min. The product was purified via HPLC. The structure of [Al(OH)(NODA*)] was verified via mass spectrometry (LC-QTOF MS) using a maXis II device (Bruker). Experiments were performed in duplicate (*n* = 2).

##### Production

**Figure 7:**
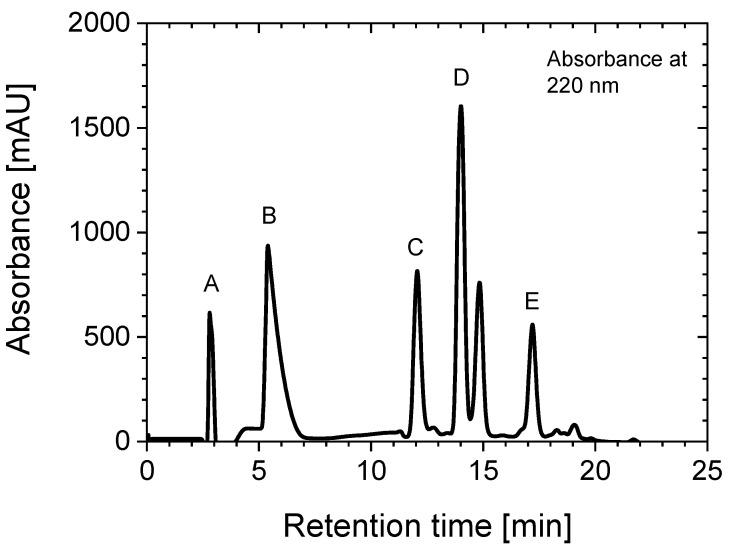
HPLC chromatogram of a crude reaction solution yielding [Al(OH)(NODA*)] (D). Other species: (C) = NODA* and (E) = impurity of the delivered NODA*. (A) = free aluminum and (B) gradient peak. Detection modes: Gamma (NaI) and UV (220 nm). Gradient: 1–25 min: 100% 0.1 M TFA aq →100% MeCN. Column: PRP-1-10^#^ (250 × 4 mm, CS-Chromatographie Service, D-52379 Langerwehe), flow: 2 mL/min. ^#^ Resin was from HAMILTON (Reno, NV, USA).

Next, 12.5 mL of AlCl_3_ standard (462.5 µmol) were reduced to 3 mL by evaporation to increase the concentration. Then, 0.6 mL of NH_4_OAc-buffer 3 M and 92.9 µmol of NODA* were added and stirred at 80 °C for 24 h. The product was isolated using a modified HPLC method (cf. Figure 7) and dried using a lyophilizer. The isolated [Al(OH)(NODA*)] was redissolved in 1.1 mL of pure H_2_O, resulting in a 0.2 mM solution stored at +4 °C under air. This solution served as a stem solution for subsequent labelling experiments with [^18^F]fluoride.

### 3.2. Synthesis of [Al ^18^F]^2+^ Chelates

#### 3.2.1. Solvent Dependency

Reaction conditions: 20 nmol of [Al(OH)(NODA*)] (10 μL) reacted with no-carrier-added (n.c.a.) [^18^F]fluoride in a 0.9% NaCl solution (35 µL) plus 5 μL of NH_4_OAc buffer 3 M pH 4.5, plus variable solvent (50 μL). Reactions with 20–40 MBq ^18^F per reaction were maintained at 105 °C for 20 min. Experiments were performed in duplicate (*n* = 2).

#### 3.2.2. Ethanol Concentration Dependency

Reaction conditions: 20 nmol of [Al(OH)(NODA*)] (10 μL) reacted with n.c.a. [^18^F]fluoride in a 0.9% NaCl solution (5 µL) plus 5 μL of NH_4_OAc-buffer 3 M pH 4.5, plus 80 µL of ethanol/water in variable ratios. Reactions with 20–40 MBq ^18^F per reaction were maintained at 105 °C for 20 min. Experiments were performed five-fold (*n* = 5).

#### 3.2.3. Temperature Dependency

Reaction conditions: 20 nmol of [Al(OH)(NODA*)] (10 μL) reacted with n.c.a. [^18^F]fluoride (35 μL) in a 0.9% NaCl solution plus 5 μL NH_4_OAc buffer 3 M, pH 4.5, plus ethanol (50 μL). Reactions with 20–40 MBq ^18^F per reaction were maintained at different temperatures for 20 min. Experiments were performed in duplicate (*n* = 2).

#### 3.2.4. pH Dependency

Reaction conditions: 20 nmol of [Al(OH)(NODA*)] (10 μL) reacted with n.c.a. [^18^F]fluoride in a 0.9% NaCl solution (35 µL) plus 5 μL of NH_4_OAc-buffer 3 M of different pH (pH < 3 was achieved by addition of HCl), plus ethanol (50 μL). Reactions with 20–40 MBq ^18^F per reaction were maintained at 105 °C for 20 min. Experiments were performed four-fold (*n* = 4).

#### 3.2.5. Time Dependency (Kinetics)

Reaction conditions: 100 nmol of [Al(OH)(NODA*)] (50 µL) reacted with n.c.a. [^18^F]fluoride (175 µL, in 0.9% NaCl solution), plus 25 µL of NH_4_OAc-buffer 3 M pH 4.5, plus ethanol (50 µL). Reactions with 20–40 MBq ^18^F per reaction were maintained at 105 °C for reaction times 0–60 min. Experiments were performed in duplicate (*n* = 2).

**Figure 8:**
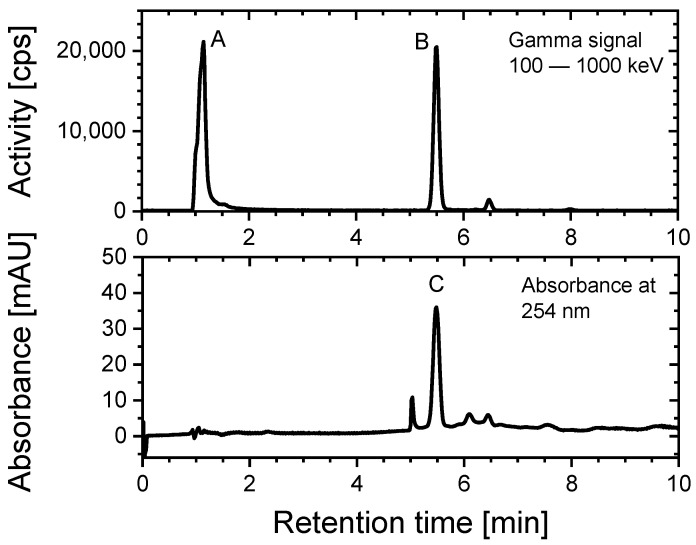
HPLC chromatogram of the labelling reaction of [Al(OH)(NODA*)] with [^18^F]fluoride. Species: (A) free [^18^F]fluoride, (B) labelled [^18^F][AlF(NODA*)] and (C) [Al(OH)(NODA*)]. Detection modes: Gamma (NaI) and UV (254 nm). Gradient: 1–19 min: 100% 0.1 M of TFA aq → 100% MeCN. Column: PRP-1-10^#^ (250 × 4 mm, CS-Chromatographie Service, Langerwehe, Germany), flow: 2 mL/min. ^#^ Resin was from HAMILTON (Reno, NV, USA).

All HPLC measurements of the labelling reaction of NODA* with [^18^F]fluoride were carried out with the same procedure. Figure 8 shows an example of these measurements. Labelled [^18^F][AlF(NODA)]* and unlabelled [Al(OH)(NODA*)] almost have the same retention time.

## 4. Conclusions

Our experimental findings confirmed that Al-NOTA* does not undergo significant ligand exchange with [^18^F]fluoride due to the high stability of the octahedral coordination of the Al^3+^ ions, and are thus in line with expectations. While Shetty at al. [27] reported low RCY in the one-pot approach, we observed negligible exchange (RCY below 0.1 %) in our two-step approach. Hence, we further focused investigations on *NODA. Difficult to explain is our finding that the complex [Al(OH)(NODA*)] was not formed quantitatively even with large excess of Al^3+^ ions. While investigating the major reaction parameters of the [^18^F]fluoride ligand exchange reaction with [Al(OH)(NODA*)], we could verify a significant increase in RCY almost by factor 2, just by substituting 50% of reaction water by a polar organic solvent, as e.g. reported by Kumar and Ghosh for a one-pot system [16]. One of the best candidates—ethanol—thereby is nicely compatible with drug formulations for human application, as already used by McBride et al. [22]. With the [Al(OH)(NODA*)] concentrations chosen in the range of 200–300 µM, the [^18^F][AlF(NODA*)] product is formed only at elevated temperatures. Furthermore, 100 °C and above were found to be optimal. Nevertheless, even with the right pH and ethanol in the aqueous reaction medium, we could not reach RCY above 80%. This outcome is already satisfying in terms of a broad application, and is basically in agreement with optimizations in a one-pot approach reported by McBride et al. [22]. However, the question remains as to why 100% RCY could not yet be achieved. One reason may be the limited thermal stability of the [Al(OH)(NODA*)] complex in the presence of sodium ions introduced with the [^18^F]fluoride. Once the Al^3+^ is released, it combines readily with present [^18^F]fluoride to form the [Al^18^F]^2+^ cation, which, in return, “sees” just a low concentration of Al-free NODA* complexes for a reaction. If this assumption was valid, the addition of metal-free NODA* right from the beginning of the second reaction step would provide a surplus of reaction partners for [Al^18^F]^2+^ and thus may help to increase the yield beyond 80%. Kinetics at 105 °C showed only moderate speed, thus making reaction times of 30 min mandatory for optimal effective yields.

In summary, the question which prompted our work, i.e., whether the one- or two-step labelling with [Al^18^F]^2+^ is advantageous, cannot be answered simply with yes or no in terms of RCY, since the RCY depends on several reaction parameters. With the chosen model chelator and the Al precomplexed NODA moiety as a labelling precursor, achievable RCY in our two-step approach are comparable with one-pot settings, as already demonstrated by D’Souza et al. [16]. The pharmaceutical advantage of using [Al(OH)(NODA*)] (+ NODA*) as a precursor is that it might make an analysis of free Al^3+^ within QC procedures needless. In terms of toxicity of macroscopic amounts of [Al(OH)(NODA*)] and NODA* in later product formulations, we do not expect problems since, e.g., in the case of common productions of carrier-added [^177^Lu]Lu-DOTATATE both, DOTATATE and Lu-DOTATATE are ingredients of the approved formulation. Further labelling investigations with aqueous [^18^F]fluoride using [Al(OH)(NODA*)] conjugated to model peptides will reveal whether this two-step approach is broadly applicable in practice. In the case of one peptide, this approach has already been demonstrated to succeed [16].

Labelling via the [Al^18^F]^2+^ cation is already now a welcome alternative to labelling with ^68^Ga^3+^, especially due to two reasons: ^18^F has the longer half-life and can be produced in very high activities by modern medical cyclotrons with moderate costs. This is of great interest for both, clinics to allow for higher patient throughputs and commercial producer of radiopharmaceuticals, leading to economically reasonable diagnostics. Moreover, approved ^68^Ge/^68^Ga generators have become very expensive, thus diminishing the rationale for ^68^Ga radiopharmaceuticals further. On top, labelling with [Al^18^F]^2+^ in aqueous media is almost as simple and robust as labelling with ^68^Ga^3+^. We believe that this Al^18^F technique will take over a significant fraction of the current ^68^Ga business.

## Data Availability

All data are contained within the article.
